# A scoping review of zoonotic parasites and pathogens associated with abattoirs in Eastern Africa and recommendations for abattoirs as disease surveillance sites

**DOI:** 10.3389/fpubh.2023.1194964

**Published:** 2023-07-17

**Authors:** Katie A. Rodarte, Jeanne M. Fair, Bernard K. Bett, Susan D. Kerfua, Folorunso O. Fasina, Andrew W. Bartlow

**Affiliations:** ^1^Genomics and Bioanalytics, Los Alamos National Laboratory, Los Alamos, NM, United States; ^2^International Livestock Research Institute and ILRI/BMZ One Health Research, Education, Outreach and Awareness Centre, Nairobi, Kenya; ^3^National Livestock Resources Research Institute, National Agricultural Research Organization, Kampala, Uganda; ^4^Emergency Centre for Transboundary Animal Diseases, Food and Agriculture Organization of the United Nations, Nairobi, Kenya; ^5^Department of Veterinary Tropical Diseases, University of Pretoria, Onderstepoort, South Africa

**Keywords:** abattoir, slaughterhouses, livestock, zoonotic disease, one health

## Abstract

Abattoirs are facilities where livestock are slaughtered and are an important aspect in the food production chain. There are several types of abattoirs, which differ in infrastructure and facilities, sanitation and PPE practices, and adherence to regulations. In each abattoir facility, worker exposure to animals and animal products increases their risk of infection from zoonotic pathogens. Backyard abattoirs and slaughter slabs have the highest risk of pathogen transmission because of substandard hygiene practices and minimal infrastructure. These abattoir conditions can often contribute to environmental contamination and may play a significant role in disease outbreaks within communities. To assess further the risk of disease, we conducted a scoping review of parasites and pathogens among livestock and human workers in abattoirs across 13 Eastern African countries, which are hotspots for zoonoses. Our search results (*n* = 104 articles) showed the presence of bacteria, viruses, fungi, and macroparasites (nematodes, cestodes, etc.) in cattle, goats, sheep, pigs, camels, and poultry. Most articles reported results from cattle, and the most frequent pathogen detected was *Mycobacterium bovis*, which causes bovine tuberculosis. Some articles included worker survey and questionnaires that suggested how the use of PPE along with proper worker training and safe animal handling practices could reduce disease risk. Based on these findings, we discuss ways to improve abattoir biosafety and increase biosurveillance for disease control and mitigation. Abattoirs are a ‘*catch all*’ for pathogens, and by surveying animals at abattoirs, health officials can determine which diseases are prevalent in different regions and which pathogens are most likely transmitted from wildlife to livestock. We suggest a regional approach to biosurveillance, which will improve testing and data gathering for enhanced disease risk mapping and forecasting. Next generation sequencing will be key in identifying a wide range of pathogens, rather than a targeted approach.

## 1. Introduction

An abattoir, commonly known as a slaughterhouse, is a facility where livestock are slaughtered for food. Abattoirs are key elements in the global food production chain and are found all over the world. Each country has unique protocols for slaughtering animals based on the size of the facility, location within communities, national and subnational regulations, and customs, including the predominant religion of the people (1). In developing countries, the raising and slaughtering of livestock is a common practice in rural areas and abattoirs (including slaughter slabs and sometimes backyard slaughters) are essential to the livelihood of the community (1, 2).

**Figure 1:**
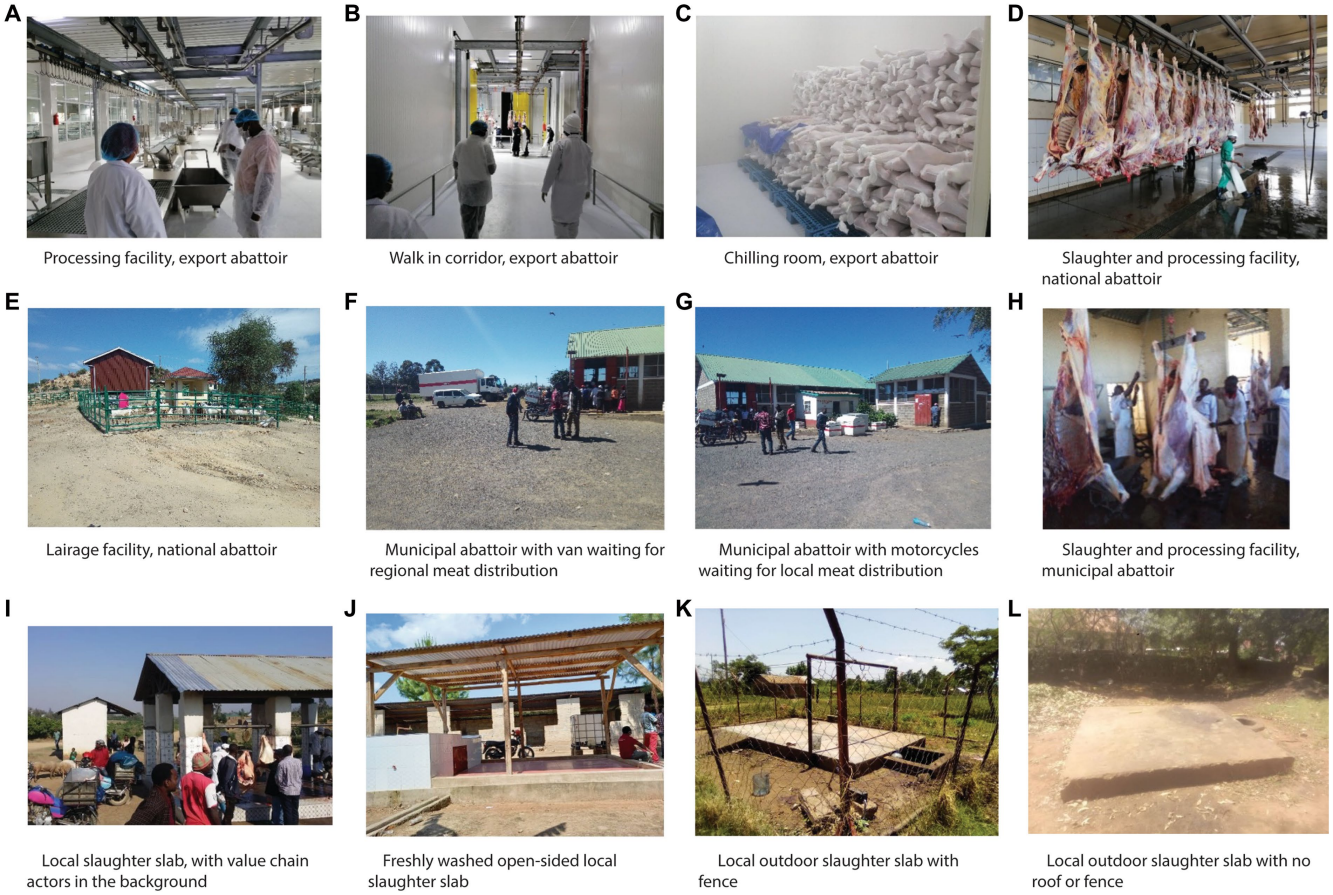
Photos showing the different types of abattoirs, including export abattoirs **(A–C)**, national abattoirs **(D,E)**, municipal abattoirs **(F–H)**, and local, backyard slaughter slabs **(I–L)**. Photo credit: Folorunso Fasina and Susan Kerfua.

Different stakeholders including butchers, traders, farm gate buyers, transporters, abattoir assistants, and water suppliers often populate abattoirs. These workers are exposed to animals, animal products, and animal waste in all types of abattoirs. There is concern that unregulated abattoirs have higher rates of occupational health problems, including zoonoses, diseases that are transmitted from animals to people, particularly because pre-slaughter and post-slaughter inspection may not be strict (3). There are four general types of abattoirs: export abattoirs, national abattoirs, municipal abattoirs, and backyard slaughter facilities or slaughter slabs (4). Figure 1 shows photos of each type of abattoir while Table 1 provides descriptions, requirements and operationalization, and associated risks. Export abattoirs are the most regulated since they are certified by national regulatory bodies and have in-house official meat inspectors. Meat is generally exported to other countries or continents, and there is low risk of infection due to state-of-the-art infrastructure and hygiene standards. National abattoirs adhere to government regulations to distribute meat within the same country. These locations have low to medium risk of pathogen transmission due to consistent veterinary meat inspections, adequate infrastructure, and sanitation practices (Table 1). Municipal abattoirs supply meat to the sub-national system, but do not comply with all national government regulations and safety requirements. These abattoirs have medium to high risk of infection because of limited infrastructure and inconsistent training, sanitation, and hygiene practices. In rural areas, backyard slaughter facilities are poorly built and often lack fencing, walls, or a roof (13) (Figure 1; Table 1). Butchering can even occur on bare ground. These facilities have a high risk of disease transmission because of minimal supervision, substandard hygiene practices and infrastructure, lack of awareness regarding disease risk, and absence of training programs (2, 13).

**Table 1:** Types of abattoirs in Eastern Africa with descriptions, requirements and operationalization, and risks associated with each one.

Abattoir type*	Description	Requirements and operationalization	Risks
Export	Typically, the export abattoirs in sub-Saharan Africa are certified by the relevant national regulatory bodies, and regularly undergo the evaluation of compliance with the Halal regulations. Such abattoir must aim to be ISO 22000, ISO 9000, and ISO 9001 certified (certification in Food Safety and Quality Management Systems). In East Africa, there are at least 44 export slaughterhouses/abattoirs (Ethiopia = 12, Sudan = 11, Uganda = 8, Kenya = 10, Tanzania = 3) and more are springing up within the sub-region (1). These types of facilities supply countries in the region or other continents once they comply with all the export requirements. These operations have both in-house and official veterinary meat inspectors and are capital intensive to set up. The facilities are comprehensive from pre-inspection to post-processing and freezing facilities. They are sited mostly in the urban and peri-urban areas.	As a requirement, all export abattoirs must comply with the following requirements in the minimum: a. Possess an Export Health Certificate from the relevant national Veterinary Service in the country. b. Have in place a Processing License from the same Veterinary Service or its outsourced agent, which is responsible for the inspection of facility to ensure that compliance with hygiene, processing standards, production quality management are met. c. Comply with the regulations guiding the issuance of Hazard Analysis and Critical Control Point (HACCP) certificate for food processors. d. Ongoing issuance of Health Certificate for every shipment by the national Veterinary Service. e. Ongoing Certificate of Origin for every shipment to be issued by the designated authorities (e.g., Revenue Authority, Customs or Chamber of Commerce and Industry). f. Meeting the importing country’s requirements for importation.	Low risk of pathogen transmission, foodborne diseases, and occupational health hazards due to: Improved awareness and understanding of the risks of foodborne disease and risk associated with operationalizing the abattoir processes. Implementation of scheduled training in HACCP, abattoir process, risk mitigation practices, reduction in contamination of animal-sourced foods. Periodic routine check for microbial risks within the operation. Adequate state of the art infrastructure and high hygiene standards Routine ante and postmortem inspections
National	These abattoirs and slaughterhouses supply major hotels, supermarkets, large butcheries, major restaurants, and food establishments requiring large quantities of standardized products. These facilities supply products within the country and are inspected by official veterinary meat inspectors. Operationalization is not as intensive compared to in the export abattoir and are mainly government owned. The facilities are also comprehensive from pre-inspection to post-processing facilities but may not always have cooling facility. They are sited mostly in the urban and peri-urban areas.	They undergo strict national government regulations aimed at improving and meeting the requirement of the national public health and safety requirements. Such regulations aim to improve hygiene and reduce the contamination of meat, reduce the risk of food borne disease, protect the consumers and protect workers from occupational health hazards. It complies with the National Meat Control Act of the country. The requirement is prescriptive of building structure and layout, equipment, personal hygiene, carcass handling, waste management, and meat inspection.	Low to medium risk of pathogen transmission, foodborne diseases, and occupational health hazards due to: Improved awareness and understanding of the risks of foodborne disease and risk associated with operationalizing the abattoir processes. Implementation of some training in HACCP, abattoir process, risk mitigation practices, reduction in contamination of animal-sourced foods. Time to time check for microbial risks within the operation. Adequate infrastructure and high hygiene standards Routine ante and postmortem inspections
Municipal	These abattoirs and slaughterhouses supply smaller quantities of standardized (and less standardized) products. These facilities typically supply products within the subnational system. Official veterinary meat inspectors inspect the facilities. Operationalization is not as intensive compared to in the national abattoir and are mainly subnational government owned. The facilities may or may not be comprehensive from pre-inspection to post-processing facilities but hardly have cooling facility. They are sited mostly in the peri-urban and rural areas.	These facilities may implement some of the national government regulations aimed at improving and meeting the requirement of the national public health and safety requirements but may not comply with all. They aim to improve hygiene and reduce the contamination of meat, but limited facilities may not always make this achievable. They aim to reduce the risk of food borne disease, protect the consumers and sometimes protect workers from occupational health hazards. The national Meat Act prescription on building structure and layout, equipment, personal hygiene, carcass handling, waste management, and meat inspection may not always be complied with.	Medium to high risk of pathogen transmission, foodborne diseases, and occupational health hazards due to: Some awareness and understanding of the risks of foodborne disease and risk associated with operationalizing the abattoir processes. Inconsistent implementation of some training in HACCP, abattoir process, risk mitigation practices, reduction in contamination of animal-sourced foods. Inconsistent check for microbial risks within the operation. Adequate (or inadequate) infrastructure and medium hygiene standards Routine ante and postmortem inspections
Backyard slaughter facility	There are poorly built and are mostly in the peri-urban and rural locations. In most cases, they are makeshift premises, which may include all kinds of places such as converted buildings or rooms, shade of trees as well as open bare grounds that a butcher or a community may find convenient for their operation. They are mostly private-owned and under no formal authority or licensing, these premises and their products are neither inspected, quantified nor subjected to trade nor health regulations. In most cases, illegal livestock trading and the slaughter of sick and diseased animals are associated with these facilities.	These facilities defy obvious norms and standards in slaughterhouse construction, equipment services and hygiene. They may have minimal supervision from official veterinary services. In selected cases, the subnational veterinary authorities pay periodic visit to these facilities.	High risk of pathogen transmission, foodborne diseases, and occupational health hazards due to: Lack of awareness and understanding of the risks of foodborne disease and risk associated with operationalizing the abattoir processes. Lack of scheduled training in HACCP, abattoir process, risk mitigation practices, reduction in contamination of animal-sourced foods. Lack of routine check for microbial risks within the operation. Inadequate infrastructure and poor hygiene standards Lack of regular ante and postmortem inspection

*In developing countries, the slaughter facilities may vary widely from large industrial meat processing facilities meeting export requirements and typically set up in the urban/peri-urban areas to small poorly unregulated facilities in the rural areas. We therefore clustered the abattoir system into four types *viz.*: Export, national, municipal and backyard slaughter facilities. Furthermore, abattoir and slaughterhouses are used interchangeably in the work. These are places where animals are slaughtered for food. There are country-level variations in designs and operationalization due to culture, animal type, investment in the industry, and country’s wealth. Sources (1, 2, 5–12).

Exposure to livestock pathogens, including bacteria, viruses, fungi, and parasites (14) can cause human morbidity and mortality. Infected livestock presents an occupational hazard to workers that encounter blood, placenta, fetuses, and uterine secretions. Human infection with pathogens at abattoirs can then lead to local outbreaks among workers (15) and can spread throughout a community either through ingestion or through direct or indirect contact (16, 17). Furthermore, each infected animal carcass that is condemned decreases the available food output for the community and reduces farmers’ incomes. Animals that are infected with dangerous pathogens can become unthrifty, die, and abort fetuses, thereby directly affecting livelihoods and economic security. For example, brucellosis, a serious bacterial disease (*Brucella* spp.) that infects livestock, can lead to low birth rates due to abortions and stillbirths (18). The extra resources that are used to prevent and limit human infection from zoonoses are an additional cost (19). Disposal of infected carcasses, for example, is a challenge because they have to be incinerated or buried. To avoid having meat condemned, animals could be immediately taken to the abattoir at the first signs of disease, for the owner to reduce the burden of losses through partial cost recovery. However, when these infected animals end up at an abattoir, workers, other livestock, and the food chain are at risk of zoonoses and trade-sensitive diseases (20).

In developing countries, inadequate veterinary infrastructure, lack of hygiene, improper meat inspection, scarcity of protective clothing, insufficient work practice knowledge, and inadequate number of staff all reduce work efficiencies and increase the likelihood of abattoir workers becoming infected. This can be a major source of foodborne illnesses, blood-borne infections, or physical injuries (21, 22). Unlike developed countries that have large industrial meat processing facilities with mandatory regulations and protocols, developing nations may have several unregulated facilities in rural areas where personal protective equipment and training is minimal or unused. In these locations, workers are more susceptible to adverse health effects, such as diarrhea, skin infections, pneumonia, meningitis, and sepsis. Some common zoonotic bacterial pathogens found in abattoirs, such as *Salmonella*, *Campylobacter*, and *Pseudomonas* (23) are becoming resistant to antibiotics and can lead to hospitalization, longer recovery time, and death. Due to the public health and economic security concerns, there is a need for governments and stakeholders to enhance abattoir infrastructure and workplace safety.

In some abattoirs, the daily supply of animals brought in for slaughter exceeds the production output. Without the organized accommodation, animal carcasses remain outside after slaughter, and are scavenged by wildlife communities. Dependent species, such as vultures and other scavenging birds (e.g., marabou stork), potentially limit infection to humans (24). However, many of these scavengers are endangered. Vulture declines are shown to lead to more feral dogs. Human interaction with these feral companions can increase the risk of zoonotic infection and lead to diseases like rabies (25). Vultures provide a crucial ecosystem service in scavenging carcasses of dead animals, and abattoirs in general have been found to be important for supporting a large number and diversity of scavenger species (26). The dissemination of carcass remains into the environment through vultures may be less risky for additional disease propagation due to scavenging species having evolved to live on decaying meat by having more acidic digestion systems than other animals (27). Understanding the system of abattoirs is coupled with understanding the avian scavenger crisis of looming extinctions and the loss of critical ecosystem functions (26).

Environmental contamination is an added risk since the abattoir wastewater and effluent may contain animal-products, pathogens (including antimicrobial resistant microbes), parasites, and residues (25, 28). Most backyard abattoirs and slaughter slabs described in Table 1 do not have the capacity and infrastructure to treat abattoir liquid waste. Therefore, liquid waste, which consists of urine, blood, and wastewater is released into surrounding areas when it rains via waterways. These pollutants can secrete into landscapes and negatively impact the environment. For example, abattoir runoff can cause harmful algal blooms (29) and result in antimicrobial resistant bacteria (30) if animal remains are not disposed of properly (31). Also, some surface waters such as streams and wells near abattoirs are highly contaminated with microbes and chemicals and become unfit for human consumption (32, 33).

In recent years, there is increased interest in abattoir-related research in Eastern Africa, but to date, we are unaware of a comprehensive review on the association of abattoirs with zoonotic disease infection in this part of the world. This scoping review will explore data collected specifically from abattoirs in Eastern Africa. The main questions for this review are (1): What parasites and pathogens have been reported from livestock and humans working at abattoirs in Eastern Africa? (2) What are other risk factors associated with these abattoirs? (3) What are things to consider for improving abattoir biosecurity and increasing biosurveillance for disease control and mitigation? In this review, we identify common zoonotic pathogens in livestock slaughtered in abattoirs and in abattoir workers, identify the sample types and tests (e.g., PCR, ELISA, culture, gross meat examination, etc.) used for pathogen detection, understand the circumstances surrounding infection risk, and identify the necessary steps to decrease disease risk at these potential hotspots. We highlight some of the growing trepidations associated with abattoirs in developing nations and propose recommendations and solutions to protect abattoir workers from occupational hazards and emerging zoonosis. We also suggest ways to improve biosurveillance at abattoirs in Eastern Africa in particular. Lastly, we cover the ecological feedback of abattoirs and their potential importance to wildlife communities.

## 2. Methods

The scope of this review was limited to abattoirs in the following Eastern African countries that are common hotspots for zoonoses: Uganda, Tanzania, Kenya, Ethiopia, Rwanda, Mozambique, Somalia, Djibouti, Madagascar, South Sudan, Eritrea, Burundi, and Zambia. We used PRISMA guidelines for scoping reviews and only included scientific peer-reviewed literature in the viable results. Web of Science was the primary database used for the search strategy. The search range consisted of data from January 1, 2010 to June 1, 2022. The following criteria was entered into the advanced search box: TS = (“slaughterhouse” OR “abattoir”) AND TS = (“zoonotic” OR “zoonoses”) AND TS = (“Ethiopia”); TS = (“slaughterhouse” OR “abattoir”) AND TS = (“zoonotic” OR “zoonoses”) AND TS = (“Tanzania”); TS = (“slaughterhouse” OR “abattoir”) AND TS = (“zoonotic” OR “zoonoses”) AND TS = (“Uganda”); TS = (“slaughterhouse” OR “abattoir”) AND TS = (“zoonotic” OR “zoonoses”) AND TS = (“Kenya”); TS = (“slaughterhouse” OR “abattoir”) AND TS = (“zoonotic” OR “zoonoses”) AND TS = (“Kenya” OR “Uganda” OR “Tanzania” OR “Rwanda” OR “Ethiopia”). TS = (“slaughterhouse” OR “abattoir”) AND TS = (“zoonotic” OR “zoonoses”) AND TS = (“Burundi”); TS = (“slaughterhouse” OR “abattoir”) AND TS = (“zoonotic” OR “zoonoses”) AND TS = (“Djibouti”); TS = (“slaughterhouse” OR “abattoir”) AND TS = (“zoonotic” OR “zoonoses”) AND TS = (“Eritrea”); TS = (“slaughterhouse” OR “abattoir”) AND TS = (“zoonotic” OR “zoonoses”) AND TS = (“Madagascar”); TS = (“slaughterhouse” OR “abattoir”) AND TS = (“zoonotic” OR “zoonoses”) AND TS = (“Mozambique”); TS = (“slaughterhouse” OR “abattoir”) AND TS = (“zoonotic” OR “zoonoses”) AND TS = (“Somalia”); TS = (“slaughterhouse” OR “abattoir”) AND TS = (“zoonotic” OR “zoonoses”) AND TS = (“South Sudan”); TS = (“slaughterhouse” OR “abattoir”) AND TS = (“zoonotic” OR “zoonoses”) AND TS = (“Zambia”).

**Figure 2:**
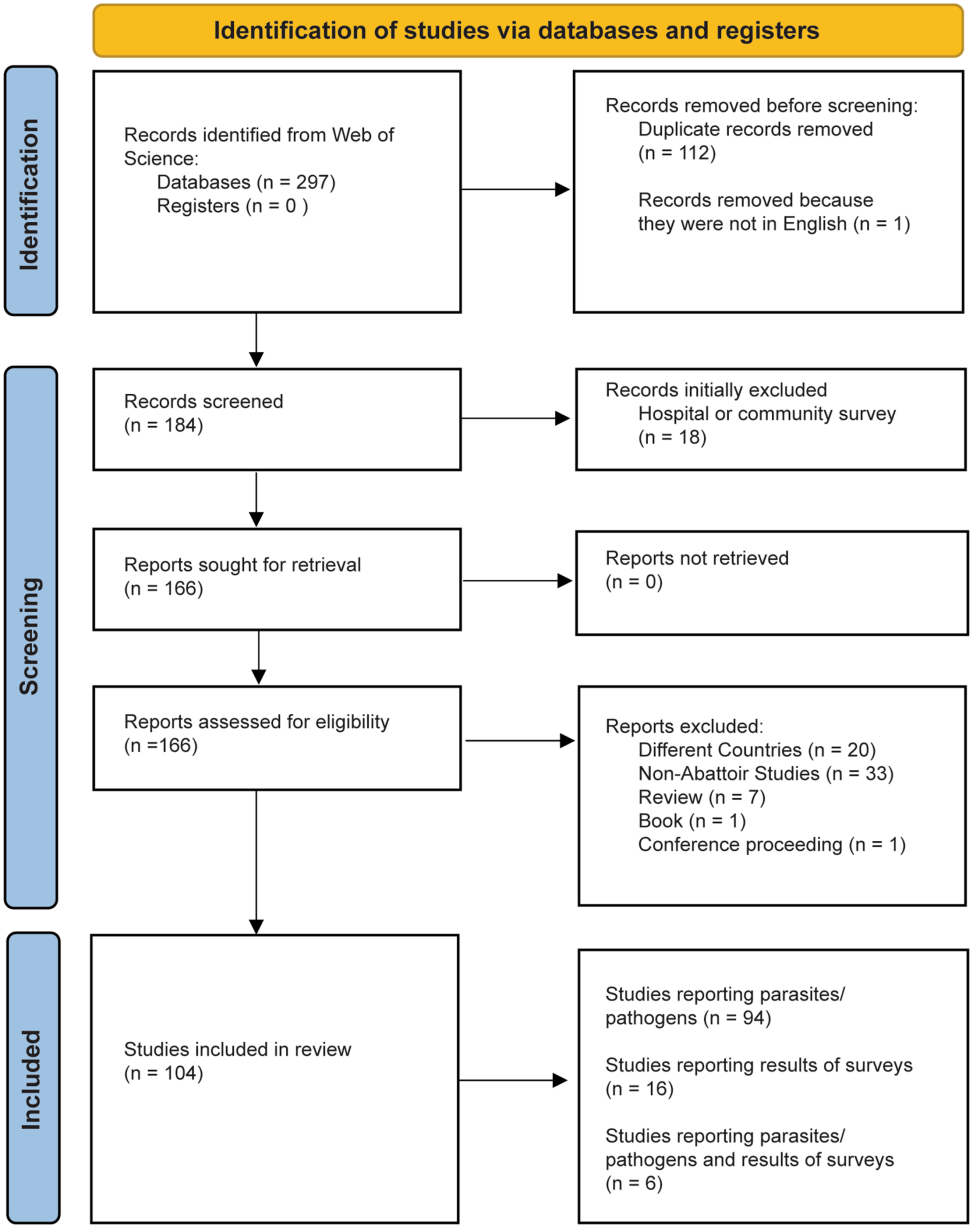
Identification, screening, and inclusion of articles in this review. Our search resulted in 104 articles.

An initial search generated 297 results. Upon first review, several of the findings were not affiliated with the location of interest, Eastern Africa, and were removed. Most studies were filtered out because they were duplicates, not conducted in abattoirs, or were done in other countries. After all the search results were adequately screened, we retrieved a total of 104 applicable articles. We kept articles that tested for and/or found parasites and pathogens in livestock and humans, as well as articles that described the results of questionnaires or surveys of abattoir workers. We removed articles that were reviewing specific diseases, but we kept results of meta-analyzes, since they reported the prevalence of pathogens from specific abattoirs. The final tally of relevant articles was 43 for Ethiopia, 20 for Kenya, 14 for Uganda, 12 for Tanzania, 5 for Madagascar, 3 for Zambia, 2 for Rwanda, 2 for South Sudan, 2 for Djibouti, 1 for Eritrea, 0 for Burundi, Mozambique, and Somalia, for a total of 104 articles (Figure 2).

The goal was not to find every pathogen detected, but to find the most common zoonotic pathogens. During the literature search, we recorded all the pathogens that were detected in each article even if they were not zoonotic pathogens. Based on our search criteria, we may have missed a few records of specific zoonotic diseases because the articles did not use the term zoonotic to refer to the specific pathogens. We could have also missed articles that did not use the terms abattoir or slaughterhouse. However, we are confident that we found the most important and common zoonotic pathogens during our search.

The final group of articles were summarized for the following data: disease and pathogens found in abattoir workers and livestock sampled at abattoirs, tests used to determine positive samples, prevalence or seroprevalence of the pathogen in humans and/or livestock, and what species of livestock was tested. We only list diseases which are caused by parasites and pathogens, not those caused by other issues, such as anthracosis (black dust in the lungs caused by breathing in carbon particles in urban areas) and melanosis. We also did not include diseases or conditions for which there could be multiple causes and the specific agent responsible was not determined (e.g., pleurisy). For the articles that reported results of questionnaires and surveys, we summarized this information and present some of the major findings regarding personal protective equipment (PPE), knowledge of disease risk, sanitation, training, and vaccination.

## 3. Results of literature survey

### 3.1. Pathogens and diseases identified in abattoirs

There were 104 articles found during the literature search that met the search criteria (Figure 2). Out of these results, 94 directly tested and found parasites and pathogens in livestock of human samples, 16 reported results of worker surveys and questionnaires (Figure 2); and 6 reported the results of both questionnaires and direct detection of the parasite or pathogen being examined. All countries except Burundi, Mozambique, and Somalia had at least one article on zoonotic pathogens in abattoirs.

Both livestock and humans were either tested or visually examined for pathogens in Eastern African abattoirs. Several articles were abattoir surveys in which they reported the results of many meat inspections and many samples taken from individual livestock animals. Therefore, these articles found more than one parasite or pathogen. Because of multiple parasite/pathogen records per article, we found 130 individual parasite/pathogen records out of the 94 articles (Table 2). These parasite/pathogen records were comprised of 42 species groups.

**Table 2:** List of parasites and pathogens found in abattoirs in countries in East Africa.

Disease	Parasite/pathogen	Zoonotic?	Country found	Positive in livestock [test(s) used]?	Positive in humans (test(s) used)?	References
African swine fever (ASFV)	African swine fever virus	No	Kenya	Yes: pigs, warthogs, ticks (PCR)	NA	(34)
Alkhurma hemorrhagic fever (AHF)	Alkhurma hemorrhagic fever virus	Yes	Djibouti	Yes: ticks feeding on cattle (PCR)	NA	(35)
Ascariasis	*Ascaris lumbricoides*	No	Tanzania	Yes: pigs (liver condemnation)	NA	(36)
Blastomycosis	*Blastomyces dermatitidis* (fungal pathogen)	No	Kenya	Yes: cattle (cellular microscopy)	NA	(37)
Bovine cysticercosis	*Taenia saginata/ Cysticercus bovis*	Yes	Ethiopia	Yes: cattle (meat inspection, morphological identification of tapeworms)	History of having human taeniasis	(38)			Ethiopia	Yes: cattle (meta-analysis)	Yes (meta-analysis)	(39)			Ethiopia	Yes: cattle (meta-analysis)	NA	(40)			Ethiopia	Yes: cattle (detection of cysts)	NA	(41)			Tanzania	Yes: cattle, sheep, goats, pigs (meat inspection)	NA	(36)			Tanzania	Yes: cattle (gross lesions)	NA	(13)			Tanzania	Yes: cattle (meat inspection)	NA	(42)			Tanzania	Yes: cattle (meat inspection)	NA	(43)			Ethiopia	Yes: cattle (post-mortem examination)	NA	(44)
Bovine pleuropneumia (CBPP)	*Mycoplasma mycoides*	No	Tanzania	Yes: cattle (CBPP lung lesions)	NA	(45)			Tanzania	Yes: cattle (CBPP lung lesions)	NA	(46)
Bovine tuberculosis (BTB)	*Mycobacterium bovis*	Yes	Ethiopia	NA	Yes (PCR)	(47)			Ethiopia	Yes: camels (post-mortem examination for lesions, PCR, spoligotyping)	NA	(48)			Ethiopia	Yes: cattle (gross lesions and culture, histopathology)	NA	(49)			Ethiopia	Yes: goats (meat inspection, culture)	NA	(50)			Ethiopia	Yes: cattle (meat inspection, culture, microscopy)	NA	(7)			Ethiopia	Yes, cattle (gross examination for lesions, Accu-Probe MTC culture identification test)	NA	(51)			Ethiopia	Yes: cattle (gross examination for lesions and culturing)	NA	(52)			Ethiopia	Yes: cattle (gross examination for lesions & Gene Probe’s AccuProbe culture identification test)	NA	(53)			Ethiopia	Yes: cattle (gross examination for lesions and microbiology tests)	NA	(54)			Ethiopia	Yes: cattle (gross examination for lesions, rapid immunochromatographic MPT64 antigen test kit)	NA	(55)			Ethiopia	Yes: cattle (gross examination for lesions, microscopy, and histopathology)	NA	(56)				Eritrea	Yes: cattle (spoligotyping, VNTR profiling, and whole genome sequencing)	NA	(57)			Kenya	Yes: cattle (postmortem meat inspection, culture, genotype MTBC assay kit)	NA	(58)			Kenya	Yes: cattle (meat inspection and culture)	NA	(37)			Kenya	Yes: cattle (meat inspection, culture, DNA strip assay kits)	NA	(59)			Tanzania	Yes: cattle (meat inspection)	NA	(36)			Tanzania	Yes: cattle (single intradermal tuberculin test)	NA	(13)			Tanzania	Yes: cattle (meat inspection)	NA	(42)			Tanzania	Yes: cattle (meat inspection)	NA	(43)			Tanzania	Yes: cattle (meat inspection)	NA	(60)			Rwanda	Yes: cattle (gross examination and culture)	NA	(61)			Zambia	Yes: cattle (meat condemnation)	NA	(62)
Brucellosis	*Brucella abortus, B. suis, B. melitensis*	Yes	Ethiopia	NA	Yes (Rose Bengal plate test and complement fixation test)	(63)			Ethiopia	Yes: caprines and ovines (Rose Bengal plate test and complement fixation test)	NA	(64)			Tanzania	Yes: cattle (Rose Bengal test)	NA	(13)			Tanzania	NA	Yes (slide agglutination test)	(65)			Uganda	NA	Yes (Microplate Agglutination Test (MAT) and Standard Tube Agglutination Test (STAT))	(9)			Uganda	Yes: pigs (positive by indirect ELISA, but negative by complement fixation test)	NA	(66)			Madagascar	Yes: cattle (qPCR, ELISA, culture)	NA	(67)			South Sudan	NA	Yes (c-ELISA)	(68)			South Sudan	NA	Yes (Rose-Bengal plate test and c-ELISA)	(69)
Campylobacteriosis	*Campylobacter jejuni* and *C. coli*	Yes	Tanzania	Yes: cattle (standard bacteriological examination-Skirrows protocol)	NA	(70)			Ethiopia	Yes: camels (culture, biochemical tests)	NA	(71)			Ethiopia	Yes: cattle, goat, chicken (standard bacteriological techniques and PCR)	NA	(72)
Contagious caprine pleuropneumonia (CCPP)	*Mycoplasma capricolum* subspp*. Capripneumoniae*	No	Ethiopia	Yes: goats, pigs (meta-analysis-microbiology and serology)	NA	(73)
Caseous lymphadenitis	*Cornebacterium pseudotuberculosis*	Yes	Ethiopia	Yes: sheep and goats (gross examination and culturing)	NA	(74)
Crimean-Congo hemorrhagic fever (CCHF)	Crimean-Congo hemorrhagic fever virus (CCHFV)	Yes	Uganda	Yes: ticks feeding on cattle (PCR)	NA	(75)			Djibouti	Yes: ticks feeding on cattle (PCR)	NA	(35)			Madagascar	NA	Yes (ELISA)	(76)
Cysticercosis	*Taenia hydatigena/Cysticercus tenuicollis*	No	Ethiopia	Yes: sheep and goats (post-mortem inspection)	NA	(77)
Echinococcosis/hydatidosis	*Echinococcus* spp.	Yes	Kenya	Yes: cattle (LAMP-LFD assay)	NA	(78)			Kenya	Yes: cattle, goats, sheep, camels (post-mortem inspection for cysts, microscopy, PCR)	NA	(79)			Tanzania	Yes: cattle (meat inspection and microscopy)	NA	(80)			Tanzania	Yes: cattle (meat inspection)	NA	(43)			Tanzania	Yes: cattle and pigs	NA	(60)			Tanzania	Yes: cattle (gross lesions)	NA	(13)			Tanzania	Yes: cattle (post-mortem inspection for cysts)	NA	(81)			Ethiopia	Yes: cattle (post-mortem examination)	NA	(82)			Ethiopia	Yes: cattle (meat inspection)	NA	(83)			Ethiopia	Yes: cattle (post-mortem examination and microscopy)	NA	(84)			Ethiopia	Yes: cattle (ELISA, IHA)	NA	(85)			Ethiopia	Yes: cattle, goats, sheep (post-mortem examination)	NA	(86)			Ethiopia	Yes: cattle (post-mortem examination)	NA	(44)			Ethiopia	Yes: cattle (gross examination for cysts)	NA	(87)			Ethiopia	Yes: sheep and goats (gross examination for lesions)	NA	(88)			Tanzania	Yes: cattle (meat inspection)		(42)
Ebola virus disease	Reston ebolavirus, Zaire ebolavirus	Yes	Uganda	Yes: pigs (ELISA)	NA	(89)
Enterococcal infection	*Enterococcus* spp.	*E. faecalis* is zoonotic	Kenya	Yes: cattle (matrix-assisted laser desorption–ionization time of flight mass spectrometry)	NA	(90)
Fasciolosis	*Fasciola* spp.	Yes	Tanzania	Yes: cattle, sheep, goats, pigs (meat inspection followed by morphological identification of flukes)	NA	(36)
			Tanzania	Yes: cattle (meat inspection)	NA	(43)			Tanzania	Yes: cattle (meat inspection)	NA	(42)			Tanzania	Yes: cattle (meat inspection and microscopy)	NA	(80)			Ethiopia	Yes: cattle, goats, sheep (post-mortem examination)	NA	(86)			Rwanda	Yes: cattle (meat inspection)	NA	(91)			Uganda	Yes: cattle (post-mortem examination)	NA	(92)
Food-borne illness	*E. coli*	No	Ethiopia	Yes: cattle (carcass swabs, Total Aerobic Plate Count)	NA	(93)			Ethiopia	Yes: camels (culture, biochemical tests)	NA	(71)			Ethiopia	Yes: sheep and goats (culturing, PCR for virulence genes)	NA	(94)			Ethiopia	Yes: goats (latex agglutination)	NA	(95)			Ethiopia	Yes, cattle (culture and PCR)	NA	(96)
Food-borne illness	*Staphylococcus aureus*	No	Ethiopia	Yes: cattle (carcass swabs, Total Aerobic Plate Count)	NA	(93)			Ethiopia	Yes: camels (culture, biochemical tests)	NA	(71)
Food-borne illness	*Klebsiella* spp.	No	Ethiopia	Yes: cattle (carcass swabs, Total Aerobic Plate Count)	NA	(93)	*Proteus* spp.	No	Ethiopia	Yes: cattle (carcass swabs, Total Aerobic Plate Count)	NA	(93)
Foot-and-mouth disease	Foot-and-mouth disease virus	No	Uganda	Yes: cattle (RT-PCR)	NA	(97)
Hepatitis E	Hepatitis E virus	Yes	Uganda	NA	Yes (ELISA)	(98)			Zambia	Yes: pigs (ELISA, nested RT-PCR)	NA	(99)			Zambia	Yes: pigs (HEV ELISA, PCR, sequencing)	Yes (EIAgen HEV Ab)	(100)
Human tuberculosis	*Mycobacterium tuberculosis*	Yes	Kenya	Yes: cattle (postmortem meat inspection, culture, genotype MTBC assay kit)	NA	(58)	*Mycobacterium* spp.	Some are	Uganda	Yes: pigs (examination for lesions, Ziehl–Neelsen staining)	NA	(101)
Leptospirosis	*Leptospira kirschneri; L. tarassovi, L. bataviae* and *L. pomona*	Yes	Kenya	NA	Yes (ELISA)	(102)			Kenya	Yes: pigs (microscopic agglutination test)	NA	(103)			Tanzania	NA	Yes (microscopic agglutination test)	(65)			Tanzania	Yes: cattle (microscopic agglutination test)	NA	(13)			Uganda	Yes: cattle (PCR)	NA	(104)			Uganda	Yes: pigs (RT-PCR)	NA	(105)
			Madagascar	Yes: cattle and pigs (PCR)	NA	(106)
Malaria	*Plasmodium falciparum*	No	Uganda	NA	Yes (Microplate Agglutination Test [MAT] and Standard Tube Agglutination Test [STAT])	(9)
Middle Eastern Respiratory Syndrome	MERS-CoV	Yes	Kenya	NA	Yes (ELISA and PRNT)	(107)
Onchocerciasis	*Onchocerca* spp.	Yes	Tanzania	Yes: cattle (meat inspection and microscopy)	NA	(80)			Tanzania	Yes: cattle (meat inspection)	NA	(43)
*Q Fever*	*Coxiella burnetii*	Yes	Kenya	NA	Yes (Serion ELISA Classic *C. burnetii* Phase 2 IgG kit)	(108)			Kenya	Yes: cattle, goats, and sheep (ELISA)	Yes (ELISA)	(109)			Ethiopia	Yes: cattle (indirect ELISA)	NA	(110)			Madagascar	Yes: cattle (ELISA) and ticks (qPCR)	NA	(67)
Rickettsiosis	*Rickettsia* spp.	Yes	Kenya	Yes: cattle, sheep, goats, ticks (qPCR)	NA	(111)			Djibouti	Yes: ticks feeding on cattle (qPCR)	Yes (ELISA)	(112)
Rift Valley fever	Rift Valley Fever Virus	Yes	Kenya	NA	Yes (Indirect ELISA)	(113)			Uganda	Yes: cattle, sheep, goats (IgM, IgG serology and RT-PCR)	Yes (IgM and IgG serology)	(114)			Madagascar	NA	Yes (ELISA)	(115)
Paracoccidioidomycosis (fungal pathogen)	*Paracoccidioides brasiliensis*	No	Kenya	Yes: cattle (cellular morphology)	NA	(37)
Pimply gut	*Oesphagostomum columbianum*	Potentially	Tanzania	Yes: cattle (meat inspection)	NA	(43)
Porcine cysticercosis	*Taenia* spp. (presumably *T. solium*)	Yes	Kenya	Yes: pigs (Ag-ELISA)	NA	(148)
Pulmonary lesions	Various bacterial pathogens (e.g., *Streptococcus* spp., *E. coli*, *Francisella*, etc.)	Some are	Ethiopia	Yes: camels (gross pulmonary lesions, culture, and biochemical tests)	NA	(116)
Salmonellosis/food-borne illness	*Salmonella* spp.	Yes	Ethiopia	Yes: cattle (carcass swabs, Total Aerobic Plate Count)	NA	(93)			Ethiopia	Yes: cattle (culturing, slide agglutination test)	NA	(117)			Ethiopia	Yes: cattle (culture)	NA	(118)
			Kenya	Yes: pigs (biochemical tests and characterized by serotyping, phage typing and plasmid analysis)	NA	(119)			Ethiopia	Yes	NA	(120)
Sarcocystosis	*Sarcocystis* spp.	Yes	Ethiopia	Yes: cattle, sheep, goats (post-mortem examination, histopathology, microscopy)	NA	(121)
Scrub typhus	*Orientia* spp.	Yes	Djibouti	NA	Yes (ELISA)	(112)
Stilesiosis	*Stilesia* spp.	No	Tanzania	Yes: sheep and goats (meat inspection)	NA	(36)
Toxoplasmosis	*Toxoplasma gondii*	Yes	Tanzania	Yes: cattle (Eiken latex agglutination test)	NA	(13)			Kenya	NA	Yes (ELISA)	(122)
Yersiniosis	*Yersinia enterocolitica*	Yes	Uganda	Yes: pigs (slow agglutination test)	NA	(66)

We list whether the diseases are zoonotic and the country in which they were found. For each article in which a pathogen was positive, we recorded whether it was found in livestock, humans, or both and what tests were used in determining positivity. If tests were not completed, then NA is listed.

Articles documented bacterial (71 records), viral (14 records), and fungal (2 records) pathogens as well as macroparasites (43 records), such as nematodes, cestodes, and trematodes (Table 2). We found two records of intracellular protozoan parasites, *Sarcocystis* spp. and *Plasmodium falciparum* (malaria). The most common pathogen detected in abattoirs was *Mycobacterium bovis*, causing bovine tuberculosis (BTB) (*n* = 22 articles), followed by *Echinococcus* spp. causing Echinococcosis/hydatidosis (*n* = 16 articles). There were 9 articles that found *Taenia saginata/Cysticercus bovis*, which causes bovine cysticercosis.

Most animals surveyed were cattle. There were 86 parasite/pathogen records in cattle. Sheep (15 records), goats (18 records), pigs (18 records), and camels (6 records) were also screened (Table 2). There was only one article that tested poultry. In Ethiopia, chickens tested positive for *Campylobacter* spp. using standard bacteriological techniques and PCR (Table 2). A high proportion of these bacteria were found to be resistant to ampicillin, amoxicillin, and streptomycin (72). There were seven instances of ticks being tested for bacterial and viral pathogens after they were removed from livestock. *Rickettsia* spp., African swine fever virus, Crimean-Congo hemorrhagic fever virus, and Alkhurma hemorrhagic fever virus were found in ticks feeding on livestock in abattoirs (Table 2). Tick sampling seems to be an overlooked method to track pathogens at abattoirs. Some zoonotic pathogens can be vectored by ticks, and sampling ticks could provide more knowledge about transmission among livestock and humans.

There were 21 instances of parasites and pathogens found in humans (Table 2). There were five articles that tested for *Brucella* spp., the most common pathogen tested for. In humans, most of the articles used various serology tests to test for past exposure. For example, tests included Rose Bengal plate test and agglutination tests for *Brucella* spp., ELISA for *Leptospira* spp., and IgM and IgG serology for Rift Valley fever virus. There was only one article that used PCR, and they tested for *Mycobacterium bovis*. One article was a meta-analysis of humans with bovine cysticercosis (*Taenia saginata*), which summarized prevalence using a variety of articles and methods. A second article was a questionnaire to determine past history of having human taeniasis, also caused by *T. saginata*, but did not directly test them in humans (Table 3). Out of 151 respondents, 71.5% reported having human taeniasis (38).

**Table 3:** Summary of the surveys and questionnaires regarding abattoirs.

Subjects being evaluated	Number of articles	References
Personal protective equipment (PPE)	9/16	(9, 102, 122–128)
Knowledge of diseases	12/16	(9, 12, 41, 68, 102, 123–129)
Training/educational programs	3/16	(123, 127, 128)
Sanitation/meat safety	6/16	(12, 125, 127–130)
Infrastructure improvement	2/16	(12, 125)
Animal trading and cross-border animal movement	2/16	(131, 132)
Consuming raw or undercooked animal products	6/16	(9, 41, 122, 124, 126, 127)
Public awareness	1/16	(123)

Listed are the number of articles that evaluated and provided results on a given subject.

Most tests used to determine animal or human infection were done using various serology tests, very few used PCR or sequencing. For example, for determining brucellosis infection, most studies used the Rose Bengal test and the complement fixation test. Most of these studies, therefore, give information regarding past exposure. For other parasites and pathogens, meat condemnation was listed, which is usually performed by a meat inspector, which looks for lesions, cysts, changes in color, and abnormal size of meat products as well as organs such as the lungs, liver, kidneys, hearts, and spleen (36). During meat inspection, a number of parasites including, but not limited to, trematodes or nematodes can be observed, and can be subsequently collected. Cysts or lesions can be sampled for further identification using molecular tools, culturing, microscopy (e.g., histopathology), and standard biochemical tests for bacteria. The following parasites and pathogens were typically found using gross examination during routine meat inspection: *Taenia saginata*/*Cysticercus bovis*, *Mycobacterium bovis*, *Echinococcus* spp., *Fasciola* spp., and *Onchocerca* spp. For some, like many bacterial species, further testing involved culturing, microscopy, and PCR tests (Table 2).

Two dimorphic fungal pathogens were detected in cattle slaughtered in a Kenyan abattoir: *Paracoccidioides brasiliensis*, which causes paracoccidioidomycosis and *Blastomyces dermatitidis*, which causes blastomycosis (37). Both of these pathogens were found using cellular microscopy. Out of 176 lesions found from 929 cattle examined, 58 tested positive for dimorphic fungi. No other studies tested for fungal pathogens. The true prevalence of these fungal pathogens is unclear and understanding livestock infection can improve surveillance efforts and environmental monitoring, which is important for understanding and tracking emerging fungal pathogens. Both fungal pathogens can infect humans, and blastomycosis has been listed as an emerging risk in both North America and Africa (133).

### 3.2. Surveys and questionnaires to assess training, sanitation, PPE, etc.

In this literature review, we found 16 articles in which surveys were conducted across Eastern Africa to assess the awareness of abattoir workers in developing nations. In each of the countries of interest in Eastern Africa, testimonials from abattoir workers demonstrated the need for disease control and implementation of prevention strategies. Through questionnaires and interviews with abattoir workers, studies found that there is an essential need for training programs, personal protective equipment, immunization programs for workers, infrastructure improvement, enhanced diagnostics and biosurveillance, and public awareness to minimize zoonosis and protect public health (Table 3).

The top subject evaluated in the surveys and questionnaires was knowledge of pathogen or disease risk from livestock (12/16 articles). Participants knew of common diseases like tuberculosis, anthrax, and brucellosis (123–125), but other diseases were less well known, such as MERS-CoV (124). Personal protective equipment (9/16 articles), consuming raw or undercooked meat (6/16 articles), and sanitation/meat safety (6/16 articles) were also asked about and evaluated in many articles (Table 2). Other subjects evaluated included infrastructure improvements, cross-border movement and trading of animals, training programs, and public awareness.

In a questionnaire survey conducted at the Harar Municipal Abattoir in Ethiopia, 300 randomly selected workers self-reported their awareness of taeniasis, caused by *Taenia saginata*, a zoonotic tapeworm parasite. Questions included awareness of taeniasis risks involved with consuming raw or undercooked meat and if workers have ever observed small tapeworm segments in their feces or clothing (41). Out of the respondents, 65% were conscious of the risks associated with *T. saginata*, including ingestion of raw or undercooked meat. Another 62% of respondents reported personal infection by the proglottids of *T. saginata* (41). The high association (*p* < 0.005) in infected participants was attributed to factors such as eating undercooked beef for religious customs, but more research is needed to determine whether the tapeworms originated from the affiliated abattoir or any of the other factors above (41). In a similar study conducted at the Kombolcha Elfora and Dessie city abattoirs in Ethiopia, 104 workers completed a questionnaire about their food safety knowledge, specifically pertaining to meat hygiene and safety techniques (129). Eighty-nine percent of participants did not know about meat safety and 74% were insufficient in their workplace practices (129).

In Kenya, public health officials offered a questionnaire to 737 participants across 142 slaughterhouses and asked about their knowledge of zoonosis, hygiene practices at the slaughterhouse, and slaughterhouse equipment practices (102). The participants also provided blood samples to check for leptospirosis, which is a pathogenic bacterial disease of the genus *Leptospira* with animal reservoirs (134). The blood test results showed a high seroprevalence of leptospirosis in 13.4% of the workers (102). Findings from the questionnaire showed that workers who were around urine or infected organs during evisceration of the carcass were at a higher risk for leptospirosis (102). Further analysis of the worker feedback showed that smoking, eating, or having an open wound created a pathway for infection within the abattoir. Slaughterhouses with roofs increased the chance of leptospirosis since the disease can live in cooler-shaded environments for longer periods of time if not adequately cleaned (102). Ingestion of waters from nearby well or spring water increased the chances of exposure due to contamination from slaughter waste or animal urine runoff.

In northern Tanzania, slaughterhouse workers, including meat inspectors, were interviewed to learn about their meat safety perceptions, priorities, and practices in relation to risk (125). The meat inspectors frequently mentioned their concern about anthrax since the disease can affect both animals and people. Several of the professionals recalled personal experiences, such as seeing visible worms in the animal intestines, and felt comfortable identifying anthrax based on the symptoms (125). Other than anthrax, many of the workers were able to identify signs of illness, such as swollen inner organs, but did not always know the name of the disease. The workers often attributed disease to unhealthy livestock keepers that may lack zoonotic awareness or are unwilling to invest in vaccination to prevent disease. When workers were asked how they know the meat is wholesome and fit for human consumption, they reported that they inspected the meat and that it was marked with a government stamp (125). For hygiene practices, all the workers knew the importance of keeping their workstation clean with soap and water but only two workers reported the importance of washing their work slab between each animal rotation. Workers reported the infrequent use of uniforms and discussed how dirty clothes, flip-flops, and lack of bathing can lead to contamination (125). Other reported contamination pathways were from the free roam of dogs, chickens, or wild birds that sometimes entered the slaughterhouse. Frequent handwashing was seldom, and meat knives were often dropped and reused after a brief wipe down (125). The same knives were used to isolate gut contents and explore feces.

In a study conducted in the Kabale District of Uganda, 348 participants from the local communities and abattoir were asked about their awareness surrounding the epidemiological risk of Rift Valley fever (126). Among the participants, 94% of butchers were aware of Rift Valley fever in comparison to 85% from other occupations (126). When the 348 participants were asked how the disease spreads, only 34 knew that Rift Valley fever can spread through mosquitos and infected bodily fluids (126). Butchers were able to identify symptoms, such as nasal discharge in animals. When asked about personal protective equipment, only 29% of participants reported usage. In this finding, butchers used the most PPE, including gumboots and aprons, most likely to reduce blood stains on their bodies and home dress, but rarely wore gloves (126). Based on the findings in Ethiopia, Kenya, Tanzania, and Uganda, there is an apparent need to increase Risk Communication and Community Engagement (RCCE) and utilize appropriate information, education, and communication (IEC) materials to create zoonotic awareness for abattoir workers and improve hygiene practices to protect public health.

## 4. Need for vaccination

Most countries demonstrated the need for vaccination to protect animals and people. For instance, one of the reasons for the continuation in the foot-and-mouth problem in Africa, includes poor performance of current animal vaccination programs (135). In a review of vaccination on the effectiveness and profitability of preventative veterinary interventions in controlling infectious diseases of ruminant livestock in sub-Saharan Africa, the authors found that vaccination is the most effective and profitable means of controlling infectious livestock diseases in sub-Saharan Africa (136). However, the authors note the challenges for disease control, and vaccination implementation must integrate pathogen surveillance and optimal vaccine delivery tools. Many, if not most, of the important zoonotic or economically devastating viral diseases in sub-Saharan Africa have developed vaccines. Controlling exposure and disease transmission in animals would reduce diseases arriving at a slaughterhouse.

In Ethiopia where there is a high human population that live in rural areas, many livestock are at the risk of brucellosis. Multiple surveys conducted in the Debre Zei and Modjo export abattoirs resulted in serological evidence of brucellosis, 1.76% in small ruminants (1.86% in caprine and 1.63% ovine brucellosis) (64). This relatively low level of seropositivity of antibodies to brucellosis may be underappreciated, and it may encourage silent spread of zoonosis to humans inadvertently. Furthermore, because *Brucella* spp. is a slow-growing organism, and people in developing countries often have poor hospitalization record (attendance), except in life-threatening situations, *Brucella* spp. infection may get established in humans, especially abattoir workers before it begins to manifest clinical signs that warrant hospitalization.

In a 9-month study conducted in 2012, a total of 566 slaughterhouse workers from 84 ruminant slaughterhouses in Kenya provided blood samples to screen for *Coxiella burnetii* antibodies, the cause of Q fever (108). The survey participants ranged from the ages of 18–82 years of age and have worked in the abattoirs or slaughterhouses for an average of 10 years. The various work roles included exsanguination through jugular severance, depilation, and skinning, eviscerating and sectioning the carcass, cleaning the intestines, and cleaning the slaughterhouse among others. An ELISA test showed that 210 workers tested positive for *C. burnetti* with a prevalence of 37.1%, which demonstrates a need for vaccinations of high-risk occupationally exposed humans (108).

In the southern Kabale district of Uganda, a March 2016 survey with 657 community members, including 117 abattoir workers, investigated participant livestock ownership along with knowledge and behaviors of Rift Valley fever (RVF) (126). Nearly half of the participants reported that they were involved with slaughtering or butchering and 69% believed they were at risk of getting RVF (126). Furthermore, 88% of butchers felt they were at higher risk than farmers or herdsman due to their contact with dead livestock (126). Although 90% of participants were aware of RVF and how people can get sick from animals, there was limited knowledge on the signs and symptoms (126). The lack of a human or animal RVF vaccine in Uganda can cause significant morbidity and mortality in humans and animals. In the four countries, the lack of vaccination remains a common denominator. If abattoir workers were vaccinated against endemic zoonotic diseases in their locality, the burden of associated morbidity and mortality may be reduced.

## 5. Effects of abattoirs in the community

### 5.1. Livestock and human infection

Parasites and pathogens can result in high morbidity and mortality in livestock resulting in the loss of animals and animal products, or reduction in the value of goods to be sold. In the case of infected females, several pathogens can cause them to abort fetuses, further limiting the ability to replace animals in the herd. The loss of livestock can impact food security in the community, increase malnutrition, and cause developmental defects like stunting in children (18). In addition, indirect effects of these infections include the fact that workers may experience loss of workdays or loss of productive years due to premature death. To minimize such disease burden in abattoirs and improve occupational health and safety within developing nations, it is essential to increase value-chain associated Risk Communication and Community Engagement (RCCE) starting from the farm and build awareness among workers.

Because abattoir serves as a link between the farm and fork, not only are abattoir workers at risk, but community members are at risk of infection if they consume infected animal products or have direct connections with abattoir workers. For instance, in a group A *Streptococcus* skin outbreak in Wales, United Kingdom, 21 workers were infected, five of which were found to have tetracycline resistant *Streptococcus* infection. In addition, four community members developed infections (16). Furthermore, other studies suggest that wind dispersal of *Coxiella burnetii*, which may be abattoir associated, can cause community outbreaks of Q fever up to 2 km away (137, 138).

### 5.2. Declines in vultures and a rise in disease

**Figure 3:**
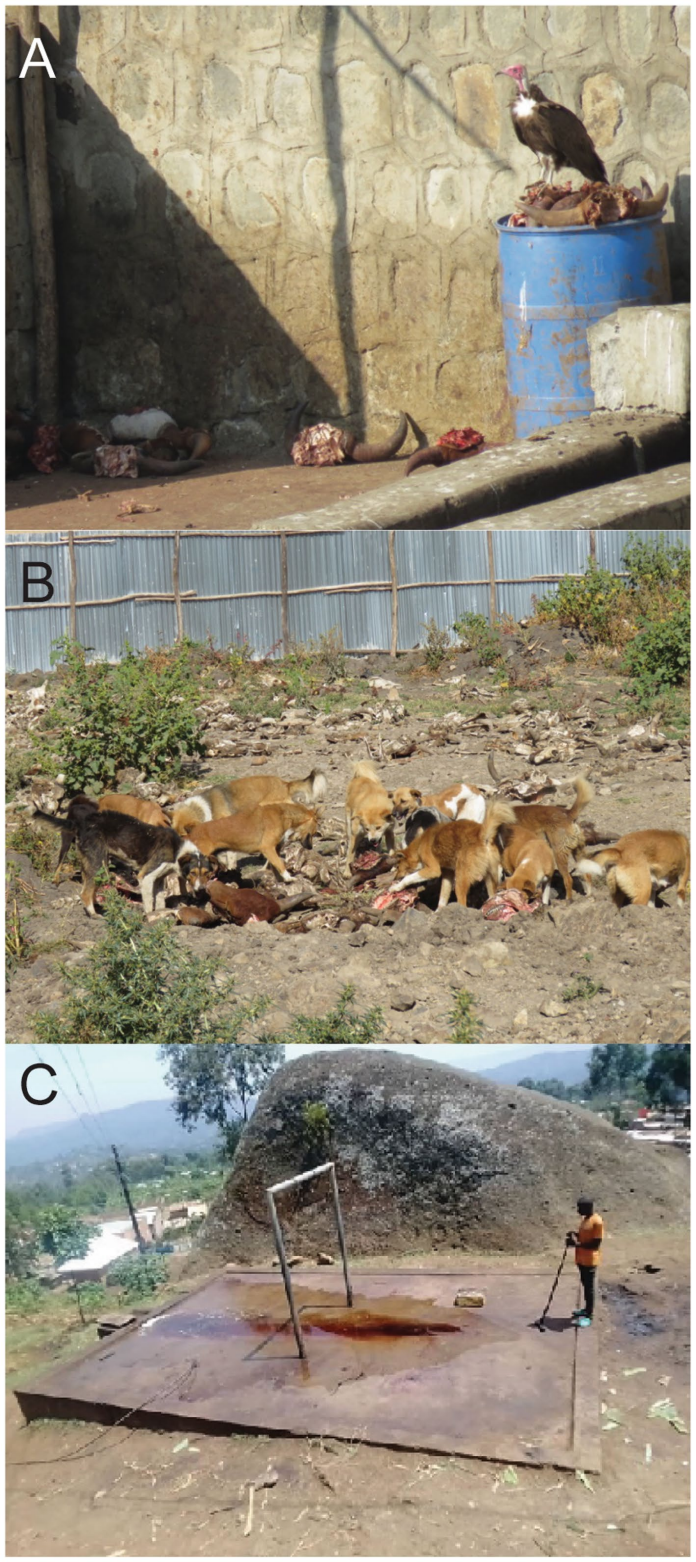
**(A)** A hooded vulture (*Necrosyrtes monachus*) and **(B)** feral dogs at an abattoir consuming slaughtered animal remains in Ethiopia. **(C)** Photo of backyard slaughter slab showing liquid waste runoff that can cause environmental contamination. Photo credits: Jeanne Fair, Susan Kerfua, Philip Wakimwere.

Backyard slaughter facilities face challenges with solid waste disposal (139). Slaughtered animal remains and other waste such as manure, bones, condemned carcasses, among others, are left at the site after a days’ work (33). This allows for vultures to scavenge. Vultures and other scavenging birds play an important role in the ecological process through scavenging and the consumption of carrion (Figure 3A). Since vultures have corrosive digestive tracts, they can quickly consume carcasses and ingest pathogens that would be harmful to other animals or people. By cleaning up animal remains in the environment, vultures help mitigate disease transmission. Declines in vultures are indirectly translating to an increase in some diseases, such as rabies. For instance, from 2014 to 2019, a study of 6 separate abattoirs in Addis Ababa, Ethiopia estimated a 12% decline in carrion consumption by vertebrate scavengers, including vultures (26) and 62% decrease in vulture population in Kenya since the 1970s. Notable species declines over the course of the study include three globally critically endangered birds: white-backed, Ruppell’s, and hooded vultures. Reasons for the decline may be attributed electrocution, poisoning, habitat loss, trapping for food, and/ or improvement in waste management practices or competitive exclusion from the rise of feral dogs (Figure 3B). Two of the abattoirs began processing carrion into animal food or fertilizers, hauling carrion to a dumpsite, or burning carrion. In the other abattoirs, there was still plenty of carrion available at the end of the study period, so food availability was not a factor. To limit feral dogs and still allow vulture access, the study suggested the addition of fences around the carrion disposal sites to reduce competitive exclusion (26). Vultures help limit the spread of pathogens by consuming infected carrion, but the observed recent declines in their population have hindered this ecosystem service.

In Eastern Africa, Marabou storks (*Leptoptilos crumeniferus*) and other scavenging birds such as piedcrow, piapiac, spur-winged lapwing, or cattle egret have become a common sight at abattoirs (140). Because some scavengers, particularly those with longer and more pointed beaks like the Marabou stork, carry their food away from slaughter sites (141), there is a risk for environmental pollution when infected animal remains drop off the stork’s beak as its moving. Nevertheless, marabou storks also play a critical role in abattoir waste management (140).

### 5.3. Environmental contamination

Environmental contamination of animal products remains a problem, even in developed countries, such as the United States and many countries in Europe. Abattoir processes result in high volumes of waste products, which need to be disposed of properly. Not only does the waste contain pathogens, but also high amounts of carbon, nitrogen, and phosphorous, which further impacts the environment. The runoff with high amounts of nitrogen and phosphorous can cause harmful algal blooms (29). In some smaller, local abattoirs, the left-over blood and animal products are left to flow into the environment and contaminate the landscape (Figure 3C). Previous studies have used wastewater to monitor infectious diseases from abattoirs including but not limited to one that identified multi-resistant *Pseudomonas aeruginosa* (23).

## 6. Designing an effective surveillance strategy at abattoirs

Due to the fact that abattoirs are the intersection between humans, livestock, and the environment, they remain potential hotspots for emerging and re-emerging diseases. Designing a targeted surveillance strategy for abattoirs is important for limiting infection and decreasing the chances of local and regional outbreaks, for both zoonotic and transboundary trade-sensitive diseases. Parasites and pathogens that abattoir workers are exposed to may be harder to control because sometimes, reservoir wildlife hosts may constantly infect cattle, sheep, and goats during grazing. Abattoirs are a ‘catch all’ for these pathogens and can potentially serve as sources of multiple infection to abattoir workers. By surveying animals in abattoirs, health officials can determine many things including, which diseases are prevalent in the different geographical locations where the animals are sourced from, whether there are changing trends and patterns, and which pathogens are most likely to be transmitted from wildlife to livestock, especially for those who grazed principally in the wildlife areas. They can ask questions like, are there regional differences in quality of meat sourced from different locations, and what are the potential roles of transport facilities along roads to the abattoirs, just to mention a few.

Abattoir-sourced epidemiological information may be beneficial. Rather than sporadic testing of people and livestock at the abattoirs, a regional approach, possibly covering areas where all animals arriving at the abattoirs are sourced from, will likely yield the best results. This includes surveying abattoirs in countries that are currently understudied like Burundi, Mozambique, and Somalia, among others. Surveillance at a regional level will allow workers and health officials to understand when and where infections are occurring to inform anticipatory actions and informed decisions. One question that relates to wildlife reservoirs and transmission to livestock is identifying the ecological factors that correlate with higher livestock diseases. For example, knowing which seasons, climate conditions, and land use types promote infection could allow abattoir workers and health officials to be more vigilant at certain times of the year, after certain climate events, or in certain habitats. Expanding this to a regional level will increase the power to effectively monitor livestock and human health for limiting disease spread throughout a community. Since these are One Health issues, having a regional network for establishing and sustaining communication, coordination, and collaboration is essential for achieving the best health outcomes.

Pathogen testing is required for early detection and successful biosurveillance. Serology is a common strategy for determining past exposure to pathogen infections or measuring antibodies to vaccination and can give a broad picture of disease risk in each area, but may not be specific for a current infection, except partially when paired serum sampling is done. PCR is a much better approach because it can determine genetic markers of pathogens and who is infected at the time of sampling. More precise conditions can be recorded as well, and aid in more accurate disease forecasting and modeling predictions. There are still drawbacks of PCR, however, which includes testing for targeted pathogens (exclusivity) that health officials are worried about or are specifically tracking, giving an opportunity to miss out on potential incidental findings. Other methods may include traditional culture for pathogens, and several versions of microscopy. There are a lot of pathogens that infect livestock and could impact the food chain and ultimately humans, but there may not be the capability or capacity to test for all things.

Intense efforts have been put over the last decade to introduce next generation sequencing and other tools in developing countries to improve biosecurity, biosafety, and biosurveillance to mitigate diseases (142). By increasing the capacity to test and do biosurveillance on more pathogens, we can get a more complete picture of the infectious disease risk in an area. We highlight the need to continue surveying abattoirs, surrounding areas, and community members who may be at the greatest risk for infection. It is likely that there are many other parasites and pathogens present at abattoirs than are currently known because of lack of testing, and higher prevalence of those currently being tested for. For example, malaria may mask other febrile infections (111) or having a fever may be diagnosed as malaria without any diagnostic test (143). Shotgun metagenomic sequencing can be done on a variety of sample types and find all important bacteria and viruses infecting livestock or humans at time of sampling.

During our literature review, we found that only three articles collected ticks and tested them for tick-borne pathogens. Tick collection can be done as livestock are brought in for slaughter. They can also be collected after slaughter as animals are being processed. Storage of ticks does not require freezing; ticks can be stored in ethanol before being processed for pathogens. Because ticks only vector certain species of bacteria and viruses, they can be screened using PCR, including new multiplex PCR panels [e.g., (144)], or sequenced using shotgun metagenomic sequencing. We suggest including tick collection in pathogen surveillance studies to better understand the circumstances surrounding transmission of tick-borne pathogens.

## 7. General abattoir recommendations

In developing countries, the governments and stakeholders should consider several proposed efforts to enhance abattoir worker safety. The first step is to improve abattoir infrastructure. In Tanzania, workers reported that scavengers, like stray dogs, were frequent visitors of the abattoir (42). Livestock remains and visceral organs are often left outside after slaughter and these carcasses become potential hotbeds for disease. Free roaming and scavenging dogs consume the infected meat and can develop parasites like tapeworms. The dogs then shed the tapeworm eggs in their feces. When grazing sheep consume the tapeworm eggs on pastures, they develop cysts in their organs from the parasites. If livestock are infected, the carcasses are condemned at the abattoir and place workers at risk. To address the problem, there needs to be a surrounding barrier to keep animals from entering the slaughterhouse perimeters.

Training programs should also be developed and implemented to educate workers about proper meat safety practices and workplace hygiene. The surveys conducted in Eastern Africa showed that most workers had little to no understanding of the implications associated with the diseases that animals can carry and transmit. Workers need to be aware that a greater proportion of emerging infectious diseases have an animal origin, and that 60% of existing human infectious diseases are zoonotic (145). Frequent handwashing, equipment sterilization, and proper cleaning of meat tables after each use will be an effective control measure against dangerous pathogens. Although there are hundreds of diseases around the world, workers should know how to identify syndromes, symptoms, and signs of infection in the animals that they work with and how to safely process infected carcasses. Once workers gain the necessary knowledge and skills surrounding meat handling, they need to have access to personal protective equipment (PPE) to safeguard from workplace injuries and illnesses. All workers should be required to wear PPE before the start of their shift. PPE should include a hard hat, safety glasses, facemask, coveralls, hard/steel toed boots, and cut resistant gloves. Earplugs should be offered to workers that use loud power tools. Other than a need for training and PPE, abattoir workers should receive vaccinations.

Vaccination could be beneficial to people that live in disease hotspots. Some vaccines that are available to humans include influenza, Q fever. Based on the work of Cook et al. (108), which screened blood samples for *Coxiella burnetii* antibodies, and another study showing 2.5% seroprevalence in community members (146), the slaughterhouse workers have a seroprevalence of 37.1%, an indication of higher risk of occupational exposure’. Although proper hand washing and the use of protective equipment can help minimize the risk of exposure, a vaccine-based approach could be an efficient means to prevent and control zoonotic infectious diseases to humans. Greater vaccine accessibility for slaughterhouse workers may be an investment but the preventative program is cheaper than the emergency response cost associated with an epidemic.

However, supplying vaccines to the appropriate places and convincing farmers and abattoir workers to get vaccinated can be difficult. Vaccine availability and supply chains are often the limiting factor for lack of vaccine use. There is also considerable hesitancy for vaccine use in smallholder farmers, which can supply abattoirs (147), including those makeshift abattoirs that have the greatest risk of transmission. One reason is that for some zoonotic diseases, which overall do not seem to significantly impact livestock before they are sold, they do not see the point of vaccinating their animals, even though they risk infection themselves (147).

Overall, the worker feedback received from surveys across several developing nations in this study demonstrates the need to protect abattoir workers from emerging zoonosis. There is an essential need for governments and stakeholders to allocate funding to increase abattoir worker awareness, require training programs, provide personal protective equipment, and encourage vaccination to minimize zoonosis and protect public health. Additional biosurveillance, including quarterly human serology testing within abattoirs, could help mitigate disease outbreaks. Although the investment could be costly, the occupational risks and emerging threat of zoonotic diseases are far too important to overlook.

## 8. Conclusion

In this scoping review, our goal was to understand the role of abattoirs for zoonotic disease risk in Eastern Africa. We identified common parasites and pathogens found in abattoirs, reviewed occupational risk factors associated with abattoirs, and provided recommendations to improve abattoir worker safety to reduce disease risk. Based on these data, we provided recommendations on how to improve biosecurity and develop a biosurveillance network in Eastern Africa. Our search results identified 42 species of parasites and pathogens in abattoir workers and livestock slaughtered at facilities found in 13 Eastern African countries The most reported pathogen was *Mycobacterium bovis*, which causes bovine tuberculosis. Recommendations to reduce disease risk include enhancing abattoir safety for workers, requiring the use of PPE, offering proper occupational training, and enforcing safe animal handling practices.

Limitations of this review include biases in the literature search process. One source of bias is not finding articles that reference zoonotic pathogens but do not refer to them as zoonotic. Another source of bias is the limitations of the articles themselves. Most articles were focused on one or two pathogens in livestock or human samples, potentially missing other important zoonotic pathogens. Additionally, many articles used serology to test for past exposure to pathogens. For livestock this is limiting for determining risk of infection to abattoir workers and communities. For abattoir workers, this information may mean that they were not infected at an abattoir; they could have been infected elsewhere. For determining risk, it is important to determine infection status at time of sampling, which can be done using PCR or next generation sequencing.

Understanding hotspots of infectious diseases should be a global priority to limit infection and prevent outbreaks. Abattoirs, particularly in developing countries, can be important tools for biosurveillance, helping to detect disease risk in a community and mitigate local outbreaks, but they are not currently being used in this capacity. It should be emphasized that abattoirs are important One Health interfaces with frequent interactions between humans, animals, and the environment, and each facility is a unique source of transmission potential that can be exploited for identifying livestock and wildlife pathogens in a community or region and aid in outbreak control and mitigation. Future work in relatively understudied countries like Burundi, Mozambique, and Somalia will improve zoonotic disease risk assessment by providing more data on important parasites and pathogens in the region. Thus, we suggest a regional biosurveillance network centered around abattoirs, which will improve testing and data gathering for enhanced risk mapping and forecasting. Next generation sequencing will be key in the ability to identify a wide range of pathogens, rather than a targeted approach that is limited in scope.

## Author contributions

JF and AB contributed to conception and design of the review. KR completed the literature search and summarized all articles. KR and AB wrote the first draft of the manuscript. JF, BB, SK, and FF wrote sections of the manuscript. All authors provided comments and edits to the manuscript, read, and approved the submitted version.

## Funding

This work was funded by the US Defense Threat Reduction Agency Biological Threat Reduction Program R-00716- 19-0 (DTRA) administered by the US Department of Defense.

## Conflict of interest

The authors declare that the research was conducted in the absence of any commercial or financial relationships that could be construed as a potential conflict of interest.

## Publisher’s note

All claims expressed in this article are solely those of the authors and do not necessarily represent those of their affiliated organizations, or those of the publisher, the editors and the reviewers. Any product that may be evaluated in this article, or claim that may be made by its manufacturer, is not guaranteed or endorsed by the publisher.
